# Pre-kidney transplant lower extremity impairment and transplant length of stay: a time-to-discharge analysis of a prospective cohort study

**DOI:** 10.1186/s12877-018-0940-y

**Published:** 2018-10-19

**Authors:** Anthony J. Nastasi, Tyler S. Bryant, Jimmy T. Le, Jennifer Schrack, Hao Ying, Christine E. Haugen, Marlís González Fernández, Dorry L. Segev, Mara A. McAdams-DeMarco

**Affiliations:** 1 Department of Epidemiology, Johns Hopkins Bloomberg School of Public Health, 615 N. Wolfe St, Baltimore, MD 21205 USA; 2 Department of Surgery, Johns Hopkins School of Medicine, Baltimore, MD USA; 3 Department of Physical Medicine and Rehabilitation, Johns Hopkins University School of Medicine, Baltimore, MD USA

**Keywords:** Kidney transplantation, Length of stay, Survival analysis

## Abstract

**Background:**

Few objective tests can be performed at admission for kidney transplantation [KT] to discern risk of increased length of stay [LOS], which is important for patient counseling and is associated with increased costs and mortality. The short physical performance battery [SPPB] is an easily administered, potentially modifiable, 3-part test of lower extremity function. SPPB score is associated with longer hospital LOS in older adults, and may provide similar utility in KT recipients given that ESRD is a disease of accelerated aging. The aim of this study was to characterize the association between SPPB-derived lower extremity function and LOS.

**Methods:**

The SPPB was administered at KT admission in a prospective cohort of 595 recipients (8/2009–6/2016). The independent association between SPPB impairment (score ≤ 10) and LOS was tested with an adjusted conventional generalized gamma parametric survival model.

**Results:**

Impaired recipients experienced longer LOS (median: 10 vs. 8 days; *P* <  0.001) with the greatest difference in percent discharged on day 10 (impaired: 54.5%, unimpaired: 73.3%). Discharge typically took 13% longer in the impaired group (relative time = 1.13; 95%CI: 1.05, 1.21, *P* = 0.001). Discharge for impaired recipients compared to unimpaired was least likely at day 5 (hazard ratio = 0.71; 95% CI:0.68, 0.74, *P* <  0.001). No differences in the SPPB impairment-LOS relationship were found by age (interaction *P* = 0.74).

**Conclusions:**

Pre-KT SPPB impairment was independently associated with longer LOS regardless of age, indicating that it is a useful, objective tool for pre-KT risk assessment in younger and older recipients that may help inform discharge planning.

## Background

Length of stay [LOS] is an important outcome associated with increased complications, death, and costs in transplant recipients [1–3]. A clear understanding of hospital LOS following kidney transplantation [KT] allows for appropriate informed consent, improved patient and caregiver counseling, as well as better discharge planning. We have recently identified transplant, recipient, and donor factors that are associated with LOS in KT recipients including pre-KT frailty [4]. Other than a frailty assessment [4–9], there is a dearth of inexpensive tests that can be performed successfully at admission for KT to identify recipients at risk of longer LOS [10].

The Short Physical Performance Battery [SPPB] is a well-validated, inexpensive, objective assessment tool of lower extremity function that was developed in community-dwelling older adults. Functional limitations, including SPPB impairment, have been shown to be associated with many important health outcomes including mortality, quality of life, functional decline, and LOS in older adults [11–14]. Our group recently found that SPPB impairment is associated with a 2-fold increased risk of post-KT mortality [15]. Given this strong association, it is likely that SPPB impairment is also associated with other KT outcomes, such as longer LOS. The SPPB measures the modifiable risk factor of functional limitations and may provide a target for interventions to prevent longer LOS.

However, there is currently no consensus regarding the most appropriate way to model the relationship between LOS and predictors of functional limitations such as the SPPB [16–18]. Conventional regression modeling treats LOS as a count of the number of days in the hospital, but does not appropriately account for patients who die during the hospitalization (non-informative censoring) and the skewed distributions of extended LOS, both of which are more common in older populations. Treating LOS as a time-to-discharge (i.e. time-to-event) analysis is seldom carried out when examining LOS, yet it allows for the calculation of informative, novel metrics of high utility for patient counseling and discharge planning [17]. This method allows for more clinically meaningful metrics to be calculated; for example, it provides the probability of being discharged each day for patients with and without SPPB impairment.

The goals of this study were to: (1) quantify the association between SPPB-derived lower extremity function and KT LOS, (2) stratify KT recipients by LOS, and (3) estimate novel and more clinically meaningful metrics of LOS by SPPB impairment using time-to-discharge analyses. We hypothesized that recipients with SPPB impairment at admission for KT would be at increased risk of longer LOS.

## Methods

### Study design

We studied 652 KT recipients who were enrolled in a longitudinal cohort study at the Johns Hopkins Hospital, Baltimore, Maryland between August 2009 and June 2016. We excluded patients whose LOS was longer than 30 days post-KT as has been done previously due to methodological and generalizability concerns (*n* = 57), leaving 595 recipients in the analysis [19, 20]. We abstracted LOS from medical records along with the recipient, transplant, and donor factors that were previously identified as potential risk factors for longer LOS [4]. Recipient factors include age, sex, race, education, body mass index, hepatitis C status, preemptive KT (i.e., KT before patient initiates dialysis), years on dialysis, previous KT, and cause of end-stage renal disease (ESRD) including hypertension, diabetes, glomerulonephritis, or other cause. Additionally, we included recipient comorbidity (as measured by the Charlson Comorbidity Index [CCI] adapted for ESRD), which was both abstracted from the medical record and self-reported [21]. Transplant factors include panel reactive antibody, ABO incompatibility, human leukocyte antigen mismatches, cold ischemic time, and donor type (live vs. deceased). Donor factors include age, sex, race, donation after cardiac death, deceased expanded criteria, hypertension, diabetes, hepatitis C status, and creatinine. The Johns Hopkins Institutional Review Board approved this study (NA_00015758) and all participants provided written informed consent.

### Short physical performance battery (SPPB)

The SPPB consists of 3 objective physical assessments (standing balance, walking speed, and repeated chair stands) of lower extremity function each with a score ranging from 0 to 4 with a summed composite score ranging from 0 to 12, as has been previously described [11]. For the balance portion, recipients were asked to stand and remain in several progressively more difficult positions (side-by-side, semi-, and full-tandem stances) for 10 s each. If a position could not be held, no further positions were attempted. For the walking speed test, recipients’ usual walking speed was timed from the first foot fall over the starting line to the first foot fall over the finish line of an 8-ft course. Finally, for the chair stand portion, recipients were asked to fold their arms across their chest and rise from a chair 5 times as quickly as possible. The SPPB was measured at the time of hospital admission for KT, prior to the start of maintenance immunosuppressive medications and inpatient dialysis; a score of ≤10 was decided a priori to signify impaired lower extremity function based on previous work looking at post-KT mortality [15].

### SPPB & LOS

We treated the LOS analysis as a time-to-event analysis and calculated the cumulative incidence of discharge using a Kaplan-Meier approach. This approach allows for a more detailed examination of discharge by impairment group with respect to time after KT and accounts for any non-informative censoring. Furthermore, using this approach we were able to calculate more clinically useful measures by estimating the changes in the relative hazard of discharge over time by SPPB impairment status as well as how many more days were needed for a given percent of impaired patients to be discharged compared to unimpaired.

We tested proportional hazards visually by graphing the log-log plot of survival and statistically using Schoenfeld residuals (*P* = 0.04), indicating the likelihood of non-proportional hazards. Therefore, we explored several parametric survival models and selected the generalized gamma [GG] model [22] based on a comparison of log likelihoods and Akaike Information Criteria. This model had the highest model quality, balancing fit and parsimony as has been previously demonstrated in LOS modeling [17]. For the GG model, we estimated 3 parameters, β (a location parameter that is related to median time to discharge), σ (a dispersion or scale parameter related to the interquartile ratio: third quartile divided by the first quartile of lengths of stay), and κ (shape). We modeled the relative hazard with time and the relative time to discharge, the factor by which times were expanded or contracted. For example, a relative time of 2 would mean that it generally took twice as long for SPPB impaired recipients to be discharged compared to the unimpaired recipients and would correspond with a relative hazard < 1.

We quantified the association between SPPB impairment and LOS after adjusting for age, sex, race, body mass index, years on dialysis, cause of ESRD, and donor type. These covariates were selected based on their relevance to LOS as well as their use in previous literature [4]. To assess the fit of this model, we graphically overlaid the GG cumulative discharge functions with the non-parametric Kaplan-Meier cumulative discharge functions for comparison. Death was not treated as a competing risk as only 2 patients died during hospitalization.

### SPPB & LOS ≥14 days

In addition, we used logistic regression models to assess both the relationship between SPPB impairment and LOS ≥14 days adjusting for the same factors as in the GG model. LOS ≥14 days was selected as an a priori outcome because it is seen as clinically relevant and has been previously used as a threshold for longer LOS after KT [4]. Using a similar approach, we assessed the independent association between a 1-point decrease in SPPB score (worse function) as well as each individual component of the SPPB (balance, walking speed, and chair stands) and LOS ≥14 days.

### Effect heterogeneity

Effect modification of the associations between SPPB impairment and LOS ≥14 days by recipient age, sex, race, diabetes status, and frailty status were tested using a Wald test. We used a similar approach to test for effect modification between as well as SPPB composite score and LOS ≥14 days.

### Sensitivity analysis of SPPB and LOS

We assessed the robustness of the adjusted associations between SPPB performance (composite score, impairment) and LOS (logistic model with LOS ≥14 days, GG model with continuous LOS) by including KT recipients omitted previously from the primary analysis for having a LOS > 30 days due to generalizability concerns (i.e., these patients not representing the typical KT recipient) as well as additionally adjusting for comorbidities including cardiovascular disease (including any ischemic heart disease, cerebrovascular disease, or peripheral vascular disease), lung disease, diabetes status, and CCI adapted for ESRD.

### Statistical analysis

A *P* value < 0.05 was considered statistically significant. All analyses were performed using Stata (version 14; StataCorp, College Station, TX) and R Statistical software version 3.3 (http://www.r-project.org).

## Results

### Study population

The mean age of the 595 KT recipients was 51.8 years (SD = 14.1, range: 18.7–86.0); 225 (37.8%) were female, 255 (42.9%) were African American, and 212 (35.6%) were live donor recipients. At time of KT, 47.1% of KT recipients were SPPB impaired. The median LOS was 8 days (IQR: 7–13, range: 2–30). Tacrolimus was associated with a shorter LOS (*P* = 0.02); no other association between immunosuppressive medication (thymoglobulin, mycophenolate mofetil, or prednisone) and LOS was found.

### SPPB at the time of admission for KT

The median SPPB score for the 595 KT recipients was 11 (IQR: 9–12, range 0–12). On average, impaired recipients were significantly older (56.3 vs. 47.8 years; *P* <  0.001), had higher body mass indices (28.1 vs. 26.7; *P* = 0.004), were on dialysis for a longer duration (3.3 vs. 2.7 years; *P* = 0.01), were more likely to be black (*P* = 0.01) and have diabetes as their cause of ESRD (*P* <  0.001) compared to those who were unimpaired (Table 1). SPPB impaired individuals were more likely to have an expanded criteria donor (11.1% vs. 4.0%; *P* = 0.01), a donor with hypertension (21.8% vs. 13.0%; *P* = 0.02), and a donor with a creatinine > 1.5 mg/dL (27.5% vs. 20.0%; *P* = 0.03). No association was found between SPPB impairment and immunosuppressive medication (tacrolimus, thymoglobulin, mycophenolate mofetil, or prednisone; all *P* > 0.35).

**Table 1:** Baseline Characteristics of Kidney Transplant Recipients by Short Physical Performance Battery (SPPB) Impairment Status, Baltimore, Maryland, 2009–2016

Factors	Unimpaired (SPPB Score > 10) *N* = 315	Impaired (SPPB Score ≤ 10) *N* = 280	*P* Value
*Recipient factors*			
Age (years)	47.8 (14.1)	56.3 (12.6)	< 0.001
Female	39.4	36.1	0.41
Race			0.01
White	55.2	41.1	
Black	36.2	50.4	
Other	8.5	8.8	
Education			0.06
High school (9–12) or less	40.6	47.0	
2-year technical school	5.5	9.7	
College/technical school	31.5	23.9	
Post-college graduate degree	22.4	19.4	
Body mass index (kg/m^2^)	26.7 (5.1)	28.1 (6.2)	< 0.01
Hepatitis C virus positive	7.0	7.6	0.8
Preemptive transplant	20.3	18.2	0.52
Years on dialysis^a^	1.7 (0.1–3.9)	2.4 (0.4–4.8)	0.01
Previous KT	20.3	15.4	0.12
Cause of end-stage renal disease			< 0.001
Hypertension	30.8	34.3	
Diabetes	10.5	21.1	
Glomerulonephritis	2.9	5.4	
Other	55.9	39.3	
*Transplant factors*			
0 Human leukoctye antigen mismatches	15.1 (14.0)	22.0 (15.5)	< 0.01
Cold ischemic time > 24 h	32.7	51.43	< 0.001
Live donor	44.1	26.1	< 0.001
*Donor factors*			
Age (years)	37.7 (13.9)	38.5 (15.4)	0.36
Female sex	49.5	43.9	0.17
Race			0.42
White	73.7	68.6	
Black	17.5	23.2	
Other	8.8	8.2	
Donation after cardiac death	19.4	22.2	0.5
Expanded criteria donor	4	11.1	0.01
Charlson Comorbidity Index^a^	0 (0–2)	1 (0–3)	< 0.001

Percentages and mean (SD) are presented unless otherwise noted

^a^Median and IQR are presented

### SPPB & LOS

**Fig. 1:**
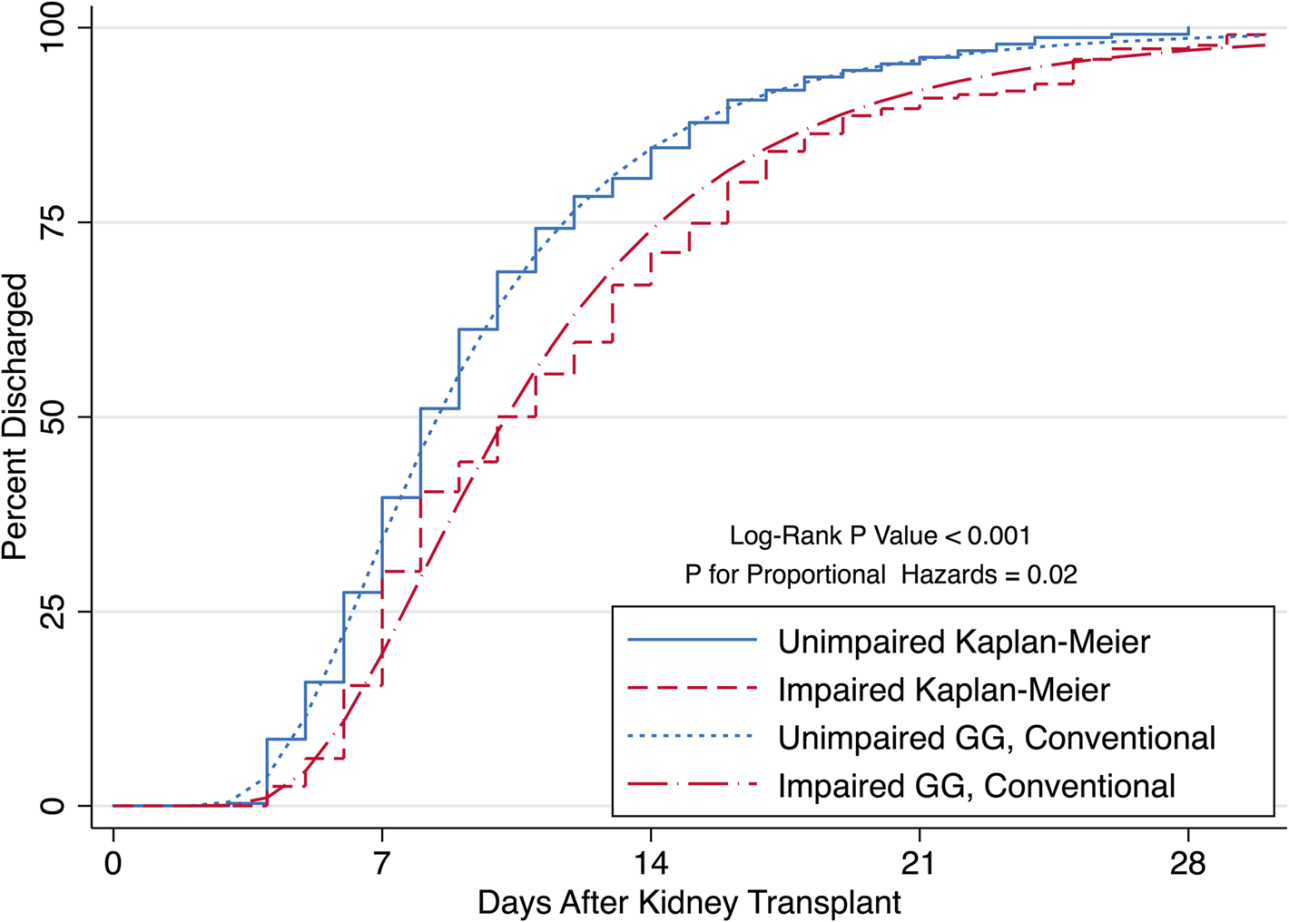
Cumulative Percent Discharged by Short Physical Performance Battery (SPPB) Impairment Status Among Kidney Transplant Recipients (*n* = 595). SPPB impairment was defined as an SPPB score ≤ 10. The Kaplan-Meier and conventional Generalized Gamma (GG) cumulative incidences of discharge are shown

SPPB impaired recipients experienced a longer LOS compared to unimpaired recipients (*P* <  0.001) (Fig. 1) such that impaired recipients had a median LOS of 10 days (IQR; 7–14) while unimpaired recipients had a median LOS of 8 days (IQR; 6–11). From the Kaplan-Meier calculations of cumulative discharge, by 7 days post-KT, 43.2% of unimpaired and 34.1% of impaired recipients were discharged (Fig. 1**,** Table 2). By 14 days post-KT, 87.9% of unimpaired and 75.3% of impaired recipients were discharged. By 21 days, 97.1% of unimpaired and 92.8% of impaired recipients were discharged. The greatest difference in percent discharged between impaired and unimpaired recipients occurred 10 days post-KT at which time 18.8% more unimpaired recipients were discharged (Table 2). The conventional GG models for impaired (β = 1.87, σ = 0.41, κ = − 0.56) and unimpaired (β = 1.75, σ = 0.41, κ = − 0.56) recipients closely mirrored the Kaplan-Meier cumulative discharge curves (Table 3, Fig. 1), confirming a good fit to describe the SPPB impairment-LOS relationship.

**Table 2:** Cumulative Percent of Kidney Transplant Recipients Discharged by Short Physical Performance Battery (SPPB) Impairment Status, Baltimore, Maryland, 2009–2016

Days Post-KT	SPPB Unimpaired^a^ (%)	SPPB Impaired (%)
1	0	0
2	0	0
3	0.3	0
4	9.2	2.5
5	16.8	6.8
6	29.8	17.2
7	43.2	34.1
8	55.6	44.4
9	65.1	48.4
10	73.3	54.5
11	78.1	60.6
12	82.2	64.5
13	84.4	71.7
14	87.9	75.3
15	90.5	79.6
16	93.0	83.9
17	94.0	87.5
18	95.2	89.3
19	95.9	91.0
20	96.5	91.8
21	97.1	92.8
22	97.8	93.2
23	98.4	93.6
24	99.1	94.3
25	99.1	96.8
26	99.4	97.9
27	99.4	97.9
28	100	98.2
29	100	99.3
30	100	100

Abbreviations: *KT* Kidney Transplant, *SPPB* Short Physical Performance Battery

^a^SPPB impairment was defined as an SPPB score ≤ 10

**Table 3:** Independent Associations of Short Physical Performance Battery Impairment with Kidney Transplant Length of Stay, Baltimore, Maryland, 2009–2016

Model^a^	Association between SPPB and LOS	
Conventional Generalized Gamma (Time-to-Discharge)	Relative Time (95% CI)	
	Impaired Vs. Unimpaired^b^	1.13 (1.05, 1.21)
Logistic Regression	Odds Ratio (95% CI)	
(LOS ≥14 Days)	Impaired Vs. Unimpaired	1.90 (1.23, 2.94)
	One-point decrease in SPPB composite score	1.16 (1.08, 1.27)

Abbreviations: *CI* Confidence Interval, *LOS* Length of Stay, *SE* Standard Error, *SPPB* Short Physical Performance Battery

^a^Adjusted for age, sex, race, body mass index, years on dialysis, cause of end-stage renal disease, and donor type. GG model parameters were β = 1.87, σ = 0.41, κ = −0.56 and β = 1.75, σ = 0.41, κ = −0.56 for the impaired and unimpaired recipients, respectively. Parameters for the GG model define the shape of the curve; β (a location parameter that is related to median time to discharge), σ (a dispersion or scale parameter related to the interquartile ratio: third quartile divided by the first quartile of lengths of stay), and κ (shape)

^b^SPPB impairment was defined as an SPPB score ≤ 10

**Fig. 2:**
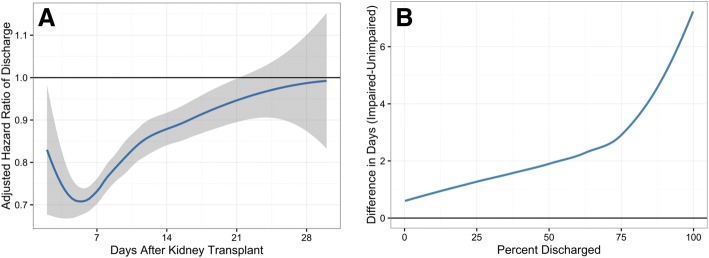
**a** Relative Hazard (HR) of length of stay (LOS) over time, comparing kidney transplant (KT) recipients who are Short Physical Performance Battery (SPPB) impaired versus those who are not. The SPPB-impaired group has a significantly lower hazard of discharge compared to the unimpaired group until around day 21, when there is no longer a significant difference between the hazards of the two groups. **b** The number of days longer that SPPB impaired kidney transplant recipients needed to reach the same percentage discharged as the SPPB unimpaired recipients. Patients who are SPPB-impaired stay in the hospital longer regardless of the percentage of patients discharged in each group. The time to reach 50% discharge was 2 days longer in the impaired group. Lower extremity impairment was defined as an SPPB score ≤ 10

The adjusted hazard of discharge was lower for impaired recipients compared to unimpaired recipients until approximately 21 days post-KT as shown by an adjusted relative hazard < 1 during this time (Fig. 2a). By day 6, the adjusted relative hazard of discharge comparing impaired to unimpaired recipients was at its lowest at 0.71 (95% confidence interval (CI): 0.68, 0.74; *P* <  0.001), indicating that impaired recipients had a 29% lower chance of being discharged on this day. The adjusted relative time comparing impaired to unimpaired recipients was 1.13 (95% CI: 1.05, 1.21; *P* = 0.001), indicating that for the same percentage of recipients to be discharged, it typically took 13% longer in the impaired group compared to the unimpaired group (Table 3). Similarly, it took approximately 1 day, 2 days, and 3 days longer for impaired recipients to achieve 25, 50, and 75% discharged compared to unimpaired recipients, respectively (Fig. 2b).

### SPPB & LOS ≥14 Days

SPPB impaired recipients experienced an adjusted 1.90-fold (95% CI: 1.23, 2.94; *P* = 0.004) higher odds of a LOS ≥14 days (Table 3). A 1-point decrease (worse function) in SPPB composite score was associated with an adjusted 1.16-fold (95% CI: 1.08, 1.27; *P* <  0.001) higher odds of a LOS ≥14 days (Table 3). Each 1-point decrease (worse function) in the balance, walking speed, and chair stand component scores were associated with 1.54-fold (95% CI: 1.27, 1.89; *P* <  0.001), 1.33-fold (95% CI: 1.08, 1.67; *P* = 0.01), and 1.23-fold (95% CI: 1.05, 1.45; *P* = 0.01) higher odds of a LOS ≥14 days, respectively.

### Effect heterogeneity

No differences in the association between SPPB impairment status and LOS ≥14 days were found by age (interaction *P* = 0.74), race (interaction *P* = 0.34), sex (interaction *P* = 0.20), diabetes status (interaction *P* = 0.71), or frailty status (interaction *P* = 0.20). Similarly, no differences in the association between SPPB composite score and LOS ≥14 days were found by age (interaction *P* = 0.67), race (interaction *P* = 0.23), sex (interaction *P* = 0.99), diabetes status (interaction *P* = 0.63), or frailty status (interaction *P* = 0.24).

### Sensitivity analysis of SPPB and LOS

SPPB impairment and composite score remained significantly associated with LOS ≥14 days in the fully adjusted logistic models that additionally adjusted for comorbidities including cardiovascular disease (including any ischemic heart disease, cerebrovascular disease, or peripheral vascular disease), lung disease, diabetes status, and CCI adapted for ESRD and included KT recipients with LOS > 30 days. Specifically, impairment was associated with a 1.74-fold (95% CI: 1.31, 2.65; *P* = 0.01) increase in risk of LOS ≥14 days; a one-point decrease in SPPB score was associated with a 1.19-fold (95% CI: 1.08, 1.30; *P* <  0.001) increase in risk. SPPB impairment also remained significantly associated with LOS in the conventional GG model such that the adjusted relative time comparing impaired to unimpaired recipients was 1.11 (95% CI: 1.03, 1.19; *P* = 0.005).

## Discussion

In a prospective, longitudinal cohort study of 595 KT recipients, we found that pre-KT SPPB composite score and lower extremity functional impairment were independently associated with increased LOS. Physical functioning in ESRD patients is important, given that ESRD is a disease of accelerated aging [10]. By modeling the relationship between SPPB impairment and LOS with a conventional GG model, we estimated that it took 13% longer for SPPB impaired recipients to be discharged compared to unimpaired recipients, and that impaired recipients experienced a consistently and significantly lower chance of being discharged until approximately 21 days post-KT. The component of the SPPB with the strongest association with LOS was the balance test in which a one-point decrease in balance score was associated with a 1.54-fold increase in odds of LOS ≥14 days. These strong, independent, and consistent findings remained in both older and younger adults, highlighting the fact that ESRD is a disease of accelerated aging with outcomes strongly tied to function abilities. Our work also demonstrates the SPPB’s great potential utility as a risk stratification tool in KT, making it one of a limited selection of tools with such potential particularly among older surgical patients.

Frailty, an aging-related syndrome marked by diminished physiologic reserve, has been studied in ESRD patients and is the main assessment tool for risk stratification in transplantation [4–9, 23–30]. Frailty is also a strong predictor of important transplant outcomes including quality of life, LOS, early hospital readmission, and mortality [4–7, 9, 23, 29–32]. The SPPB, a measure of functional limitation, consists of completely objective components and is less burdensome to measure than frailty given that it consists of fewer components and requires no additional tools. These qualities facilitate its use in non-research, clinical settings, permitting improved patient counseling and other clinical goals for which frailty may not be as easily administered. Furthermore, transplant centers tend to have their own procedures for evaluation, waitlisting, and pre-operative counseling, often relying on comorbidities and age. The SPPB, therefore, offers a standard, reliable tool across centers.

Functional limitation as measured by SPPB has been found to be predictive of important health outcomes in a variety of populations [11, 33]. To our knowledge, three other works have looked at the SPPB in transplant populations. Nastasi et al. and Wang et al. found an association between pre-KT or pre-liver transplant SPPB score, respectively, and waitlist death/delisting [34]. Lorenz et al. also found an association between pre-KT SPPB and LOS; however, this association was not explored in depth and was limited to a logistic regression of LOS > 4 days [35]. Our findings extended this work to KT recipients in demonstrating a robust association between SPPB-measured lower extremity impairment and post-KT LOS.

Functional limitations is associated with worse outcomes in patients across the kidney disease spectrum. Longitudinal studies in aging have shown that functional limitation is a critical step in the pathway describing progression from disease to disability [36, 37]. This has been observe in patients with ESRD, where the trajectory of recovery is rapidly downward without efforts to improve or preserve physical functioning. For example, of the 366 patients who received a transplant within 24 months of initiating dialysis in the Dialysis Morbidity and Mortality Study, those with a lower self-reported physical functioning before KT were more likely to be rehospitalized after transplantation [38].

Functional limitation, however, can be modified through prehabilitiation. There is evidence that patients with higher physical functional capabilities before a surgical intervention will better tolerate a procedure such as KT. For example, a recent review of the literature by Cheng et al. identified a growing body of evidence documenting improved outcomes in cardiopulmonary fitness with exercise training in patients after cardiothoracic and orthopedic surgeries, including post-cardiac transplantation [39]. There is also evidence that most nephrologists, geriatricians, transplant surgeons, and ESRD patients agree that prehabilitation could make ESRD patients less frail [40]. Because functional limitations can be improved through intervention, pre-KT risk assessment with the SPPB may provide an opportunity to intervene by encouraging patient participation in a prehabilitation program.

To our knowledge, this was the first use of a parametric GG survival model to analyze LOS in a surgical population and to calculate novel LOS metrics, which provide a detailed and informative characterization of the differing trajectories of recipients with functional limitations over the course of hospital stay. These methods, in addition to being informative regarding clinical utility, appear to be the most appropriate methodologically when considering predictive ability and model quality [16, 17]. These are likely even more important methodologic weaknesses to consider when looking at older surgical populations because they take into account noninformative censoring (e.g., death) and better handle skewed distributions of extended LOS, issues likely more common in older adults. Using our methodologically appropriate approach, a recipient can know their absolute and relative chance of discharge on any given day post-KT, which provides the chance to better inform care of older adult surgical patients.

A limitation of our work is that we only had data from a single transplant center, which may limit generalizability of our findings. However, we believe the participants in this study are at least representative of the entire KT population at our institution as our cohort of participating KT recipients were not significantly different from those that did not agree to participate and likely represent the KT population in general. Additionally, we found no effect heterogeneity by major recipient characteristics, further suggesting that our findings are generalizable to other populations that might not share the same characteristics as ours.

This study has several important strengths including its prospective design, the representativeness of the cohort, and the measurement of the SPPB, a novel measure of functional limitation. Ascertainment and assessment of recipient factors in this cohort are strengths given that the national registry data of KT recipients lack granular measurements of specific gerontology concepts like the SPPB.

## Conclusions

Pre-KT SPPB impairment and composite score were both independently associated with increased LOS, suggesting that the SPPB is a high-utility objective physical assessment capable of successful post-KT risk stratification for LOS and potentially other important KT outcomes. Furthermore, the use of a parametric survival model like the GG model allowed for a more informative and methodologically appropriate analysis of LOS and should thus be considered in place of conventional regression approaches especially in older adult surgical populations given their unique methodologic considerations. Based on these findings, SPPB impaired individuals should be identified at time of KT to facilitate successful patient discharge counseling and to potentially receive more careful follow-up to avoid adverse events associated with an increased LOS.

## Abbreviations

CI: Confidence interval
ESRD: End-stage renal disease
GG: Generalized gamma
KT: Kidney transplantation
LOS: Length of stay
SPPB: Short Physical Performance Battery
